# Bacillus Calmette–Guérin (BCG)‐associated hemophagocytic lymphohistiocytosis in the setting of IFN‐γR1 deficiency: A diagnostic dilemma

**DOI:** 10.1002/jha2.5

**Published:** 2020-04-28

**Authors:** Anahita Razaghian, Leila Parvaneh, Mona Delkhah, Arash Abbasi, Parisa Sadeghirad, Mohammad Shahrooei, Nima Parvaneh

**Affiliations:** ^1^ Department of Pediatrics Division of Allergy and Clinical Immunology Tehran University of Medical Sciences Tehran Iran; ^2^ Department of Biology Central Tehran Branch Islamic Azad University Tehran Iran; ^3^ Flow Cytometry Laboratory Children's Medical Center Tehran Iran; ^4^ Department of Pediatrics Tehran University of Medical Sciences Tehran Iran; ^5^ Department of Microbiology and Immunology Laboratory of Clinical Bacteriology and Mycology KU Leuven Leuven Belgium; ^6^ Research Center for Immunodeficiencies Tehran University of Medical Sciences Tehran Iran

## Abstract

Hemophagocytic lymphohistiocytosis (HLH) disease is a severe immune dysregulation caused by mutations in genes required for lymphocyte cytotoxicity function. However, HLH‐like syndrome may develop secondary to infections, malignancy, and autoimmunity. Primary immunodeficiencies (PIDs) could predispose to HLH syndrome after uncontrolled infections. Mendelian susceptibility to mycobacterial disease (MSMD) is a PID characterized by a predisposition to clinical disease caused by weakly virulent mycobacteria, such as bacillus Calmette–Guérin (BCG). Inborn errors of interferon‐γ immunity caused by mutations in 16 genes, underly MSMD development. Here, we report a case of fatal interferon‐γ receptor 1 deficiency with disseminated BCG infection, which was initially diagnosed with HLH disease. We also include a review of cases reported in the literature.

## 1 CASE REPORT

A 3‐month‐old female born to consanguineous parents was referred due to fever, pallor, rash, hepatosplenomegaly, and left axillary lymphadenopathy. She had received bacillus Calmette–Guérin (BCG) vaccine at birth. At 2 months, she had presented with fever, hepatosplenomegaly, and bicytopenia (hemoglobin 5.5 g/dL, white blood cells 14,220/μL, absolute neutrophil count 8,130/μL, lymphocytes 3,924/μL, and platelets 38,000/μL). Bone marrow study at that time was inconclusive; however, based on high ferritin (2800 ng/mL; normal range 100–400 ng/mL) and triglyceride (273 mg/dL; normal range 40–160 mg/dL), a provisional diagnosis of hemophagocytic lymphohistiocytosis (HLH) was made. Induction therapy with etoposide and dexamethasone (HLH‐94 protocol) started, and she was referred to us for hematopoietic stem cell transplantation.

A thorough workup to study underlying immunodeficiency performed. Broad‐spectrum antibiotics started, and dexamethasone continued. Blood culture was negative. Aspiration of axillary lymph node and bone marrow biopsy were positive for acid‐fast bacilli. The molecular study confirmed infection with BCG. No hemophagocytic cells documented in the bone marrow. Antimycobacterial chemotherapy (Isoniazid, Rifampin, Ethambutol, and Amikacin) and subcutaneous interferon‐gamma prescribed (100 μg/m^2^/day).

**FIGURE 1:**
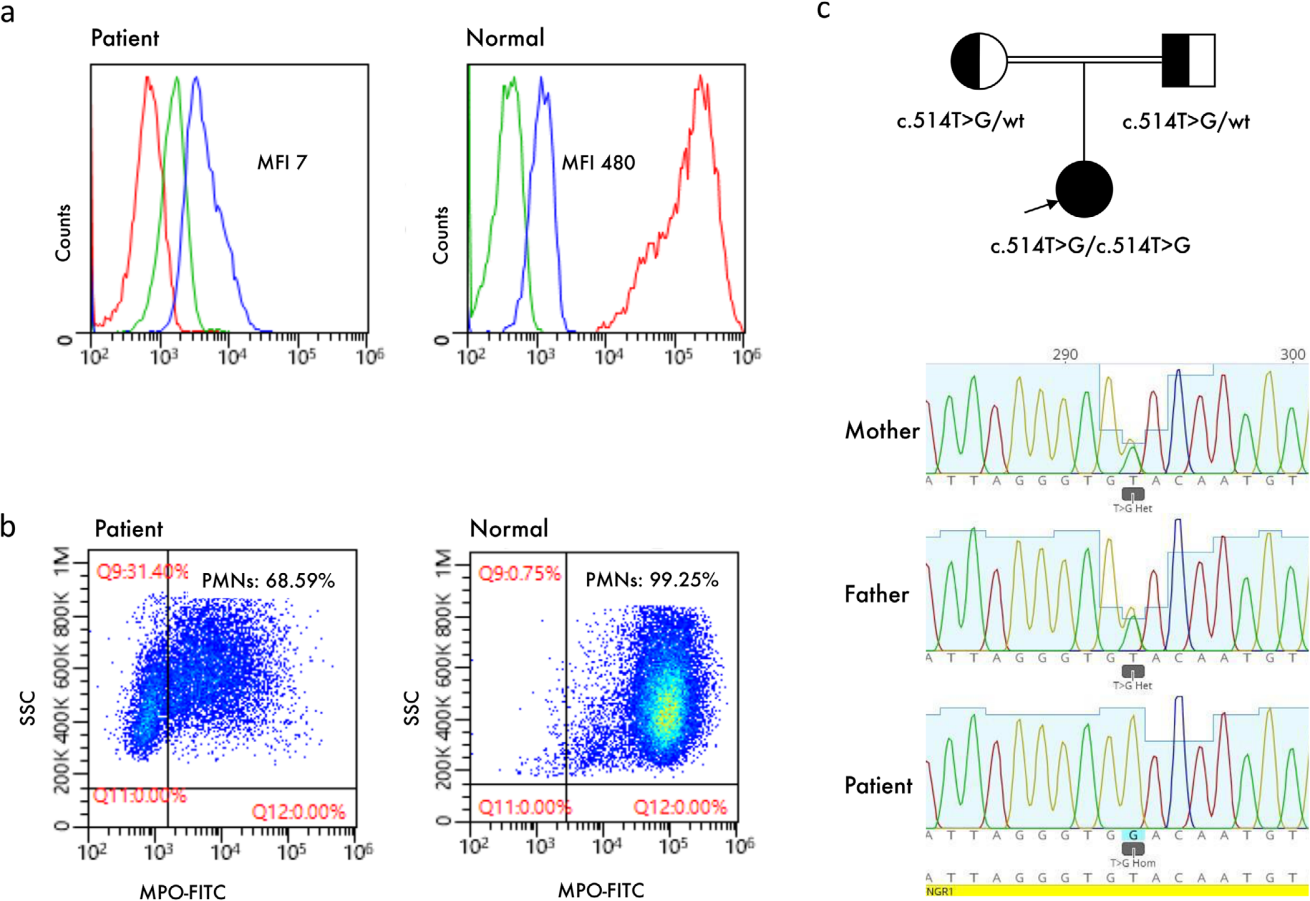
(A) DHR plots from the patient (left) and control (right); (B) MPO expression in neutrophils of the patient (left) compared to control (right); (C) Pedigree of the family and the familial segregation of *IFNGR1* mutation.

The immunologic studies showed abnormal neutrophilic oxidative burst using flowcytometric dihydrorhodamine (DHR) test; however, nitroblue tetrazolium (NBT) test was normal. Myeloperoxidase (MPO) expression modestly decreased in peripheral neutrophils (Figure 1). Her clinical condition deteriorated during the next 2 weeks with *Staphylococcus epidermidis* sepsis and progressive cholestasis. Finally, she died of multiorgan failure at 4 months. Whole exome sequencing (WES) showed a homozygous mutation in *IFNGR1* (NM_000416; c.514T > G) coding for interferon‐γ receptor 1(IFN‐γR1) (Figure 1). This mutation causes a missense amino acid change at highly conserved codon 172 (p.Y172D).

This mutation has not been reported in the Human Gene Mutation Database (http://www.hgmd.cf.ac.uk/ac/all.php) and ClinVar Miner (https://clinvarminer.genetics.utah.edu). Sorting Intolerant From Tolerant (https://sift.bii.a-star.edu.sg) and PolyPhen‐2 (http://genetics.bwh.harvard.edu/pph2/index.shtml) tools predicted this variant as deleterious. Moreover, this variant had a Combined Annotation Dependent Depletion score of 23 (https://cadd.gs.washington.edu) that is highly pathogenic.

Parents were heterozygous for this mutation. As the genetic result became available postmortem, no cytokine production assay performed. No mutations in *MPO* and *CALR* (encoding calreticulin) underlying inherited MPO deficiency documented in WES results.

## 2 DISCUSSION

HLH syndrome is an inflammatory process with a distinctive pattern that develops in different clinical settings. Only a subset of these patients in whom cytotoxic granule exocytosis is defective is considered HLH disease (primary HLH) [1]. On the other hand, some primary immunodeficiencies (PIDs) have reported presenting with HLH within the context of severe unresolved infections [2]. Hemophagocytic lymphohistiocytosis disease typically requires punctual recognition and treatment; though, HLH‐directed chemotherapy may be harmful to a patient with undiagnosed PID. Besides common HLH‐associated viruses, other infections such as visceral leishmaniasis, mycobacteria, fungal, and bacterial infections can trigger HLH. In these cases, direct treatment of the infection is favored to HLH‐directed therapy [3]. Here, we present a patient who developed BCG‐associated HLH due to underlying IFN‐γR1 deficiency, though making a diagnostic challenge. The patient presented with an interim diagnosis of HLH disease and already had received several infusions of cytotoxic drug etoposide and dexamethasone. The presence of disseminated BCG infection mandated a thorough study to decipher underlying PID. Short of WES genetic verification, immunologic workup showed abnormally reduced DHR in the presence of a normal NBT (Figure 1). A low neutrophil DHR signal can be seen in both chronic granulomatous disease (CGD) and MPO deficiency. In contrast to the case in DHR, the NBT assay is insensitive to MPO deficiency and distinguishes CGD (abnormal NBT test) from MPO deficiency (normal NBT test) [4]. The WES study excluded genetic causes of MPO deficiency, putting drug‐induced MPO deficiency as the best explanation for abnormal laboratory findings in the patient. At last, the WES study confirmed IFN‐γR1 deficiency as the underlying defect.

Autosomal recessive IFN‐γR1 (OMIM*107470) deficiency is the first identified genetic etiology of Mendelian susceptibility to mycobacterial disease (MSMD). The most severe types of complete receptor deficiency present with early‐onset, disseminated, and life‐threatening mycobacterial disease, whereas the partial types have a late‐onset and more favorable picture [5]. Other viral and rarely bacterial infections have also been described [6]. The overall prognosis is poor, with about 60% of mortality among reported patients [7]. Our patient presented with HLH‐like syndrome after BCG infection. Though tuberculosis has been increasingly reported as a potential cause of HLH, nontuberculous mycobacteria infections associated with HLH are still very rare [8, 9]. Bacillus Calmette–Guérin‐associated HLH very rarely complicates intravesical BCG instillation aimed to treat superficial bladder cancer [10]. Severe BCG disease specifically complicates some types of PID, such as severe combined immunodeficiency and CGD, but interestingly, there is a lack of HLH‐like syndrome reported in the literature [11, 12]. Disseminated BCG infection shares many clinical and laboratory features with HLH. It would be sensible to obtain HLH criteria in PID patients with disseminated BCG. A literature review showed six other MSMD cases presenting with HLH during their follow‐up (Table 1) [13, 14, 15, 16]. All of them fulfilled the current criteria for HLH, and HLH episodes were mostly associated with a viral infection. Interestingly, hemophagocytic cells were not detected in all but one of the patients, showing the low sensitivity of this criterium. Those who triggered by a mycobacterial infection (solely or in addition to a viral infection) and received HLH‐directed chemotherapy had a poor outcome.

**TABLE 1:** HLH characteristics in MSMD patients

	Patient 1	Patient 2	Patient 3	Patient 4	Patient 5	Patient 6	Patient 7
**Sex**	Female	Female	Male	Female	Male	female	Female
**Origin**	Portugal	China	Mexico	Mexico	Mexico	Morocco	Iran
**Gene**	*IFNGR2*	*IFNGR1*	*IL12RB1*	*IL12RB1*	*IFNGR1*	*IFNGR1*	*IFNGR1*
**Mutation**	c.74‐216del, p.D25Afs*38, Homozygous	c.655G > A, p.G219R, Homozygous	c.182A > G, p.E61G, Homozygous	c.700_701ins TTGGTTTG GTTCTGAT TGCAG, Homozygous	c.818del4, Heterozygous	p.W99R Homozygous	c.514T > G, p.Y172D, Homozygous
**HLH onset age**	2 months	4 years	5 years	14 years	18 months	4 months	2 months
**Infectious triggers**	CMV, *M. bovis*	EBV, *M. tuberculosis*	nd	nd	EBV	BCG	BCG
**HLH criteria**	7/8	5/8	5/8	5/8	5/8	6/8	5/8
**Fever**	+	+	+	nd	+	+	+
**Splenomegaly**	**+**	**+**	**+**	**nd**	**+**	**+**	**+**
**ANC (cell/μL)**	2.5	6.7	≈ 8	nd	5.8	Normal	8.13
**Hb (g/dL)**	**6.7**	**7.7**	**nd**	**nd**	**5**	**5**	**5.5**
**Plt (cell/μL)**	10000	21000	nd	nd	213000	30000	38000
**Ferritin (ng/mL)**	5434	35292	↑	↑	5540	1043	2800
**Triglyceride (mg/dL)**	1346	655	↑	↑	366	187	273
**Fibrinogen (mg/dL)**	**90**	**270**	**↓**	**↓**	**nd**	**420**	**398**
**sCD25 (U/mL)**	**>200000**	**nd**	**nd**	**nd**	**nd**	**nd**	**nd**
**Hemophagocytosis**	‐	‐	‐	‐	+	‐	‐
**Treatment**	Dexamethasone, CsA, ATG	Dexamethasone, Etoposide, ATG, anti‐CD20, 5‐drug antitubercular therapy	Steroid, CsA, IVIG	Steroid, CsA, IVIG	HLH‐2004	Steroids, CsA, ciprofloxacin, amikacin, clarithromycin, RIF, INH, EMB	HLH‐94, INH, RIF, amikacin, EMB, rIFN‐γ
**HLH outcome**	Deceased	Deceased	Resolved	Resolved	Resolved	Resolved	Deceased
**Reference**	[16]	[16]	[14]	[14]	[15]	[13]	Current case

**Abbreviations**: ANC, absolute neutrophil count; ATG, antithymocyte globulin; CsA, cyclosporine; EMB, ethambutol; INH, isoniazid; IVIG, intravenous immunoglobulin; nd, not determined; RIF, rifampin.

The pathogenic mechanisms underlying HLH syndromes remain unclear. Current knowledge on the pathogenesis of HLH is coming from mice. Increased proinflammatory cytokine (eg, IFN‐γ and IL‐18) release and Toll‐like receptor 9 (TLR9) overstimulation have been implicated; however, none of them have been substantiated [17, 18]. Development of HLH in patients with IFN‐γR deficiency highlights the significance of IFN‐γ–independent mechanisms in the pathogenesis of at least a proportion of HLH manifestations. Indeed, excessive production of IFN‐γ leads to hematologic features (eg, hemophagocytosis), whereas disproportionate consumption of IL‐2 contributes to immunologic features and disease progression typical to HLH [13].

Hemophagocytic lymphohistiocytosis in the setting of MSMD could be triggered by a viral and/or mycobacterial infection. The current HLH diagnostic criteria are insufficient to discriminate these patients from primary HLH disease. Timely and accurate diagnosis of triggering infection is mandatory, as the utility of HLH‐directed therapy seems to be controversial and even detrimental.

## AUTHOR CONTRIBUTIONS

A.R., L.P., and A.A. wrote the paper, M.D. performed FACS studies, P.S. followed the patient, M.S. performed genetic studies, and N.P. designed the study, followed the patient, and wrote the paper.

## CONFLICT OF INTEREST

The authors declare that there is no conflict of interest.
